# Supplementary figures and images for: Assessing bnAb potency in the context of HIV-1 envelope conformational plasticity
Source: PLoS Pathog. 2025 Jan 21;21(1):e1012825. doi: 10.1371/journal.ppat.1012825 (PMC11774494; doi:10.1371/journal.ppat.1012825)

S1 Fig

A.

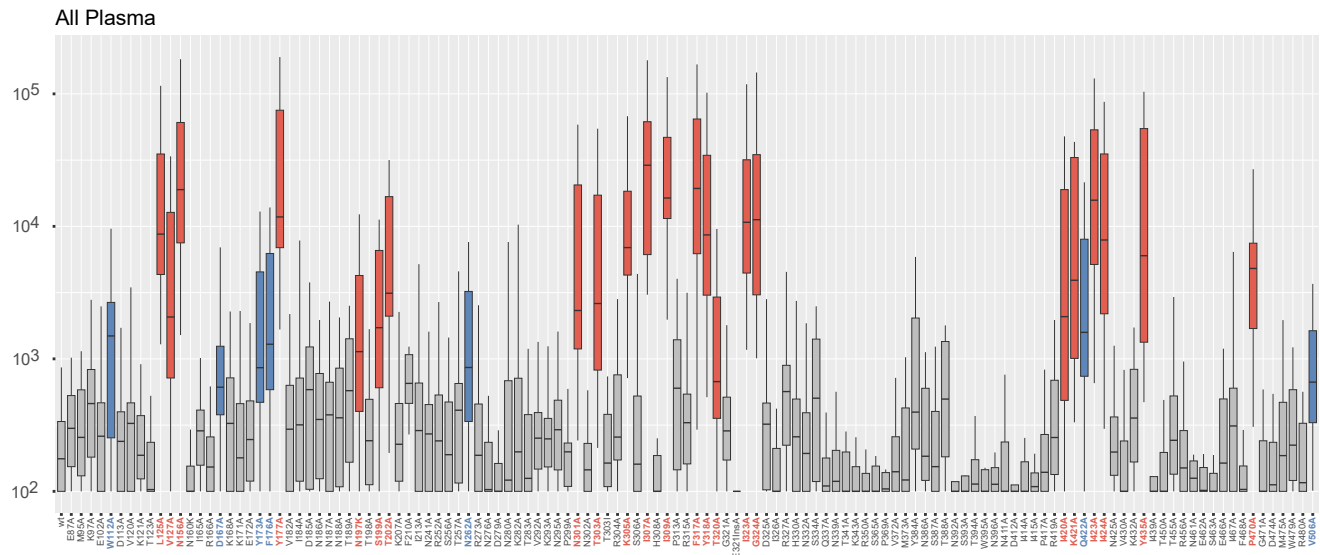

B.

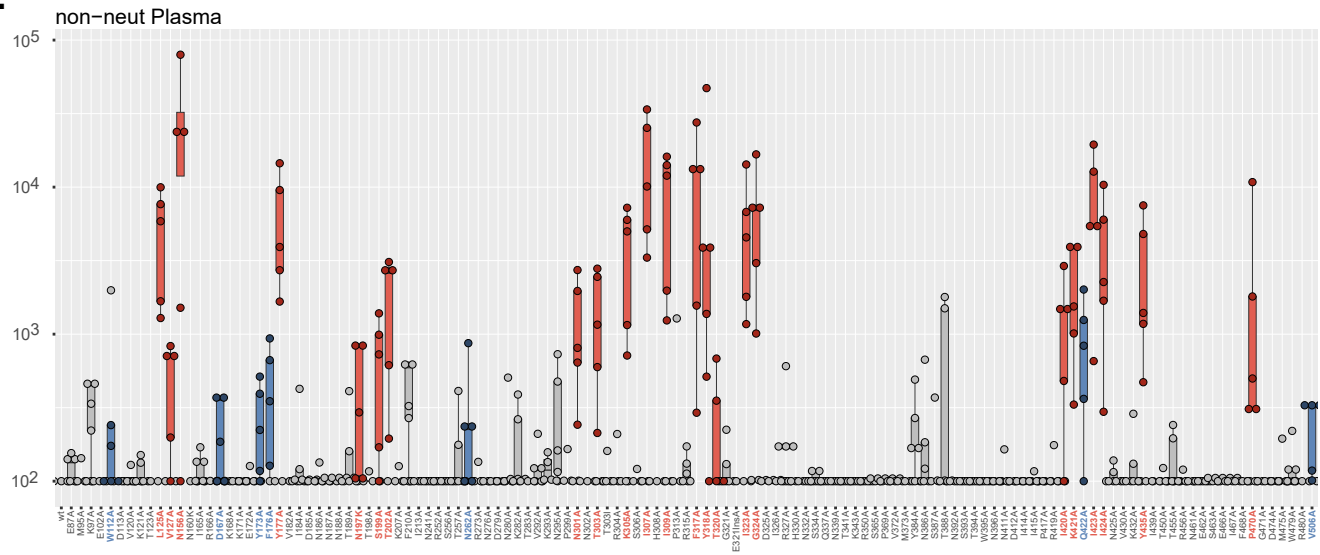

C.

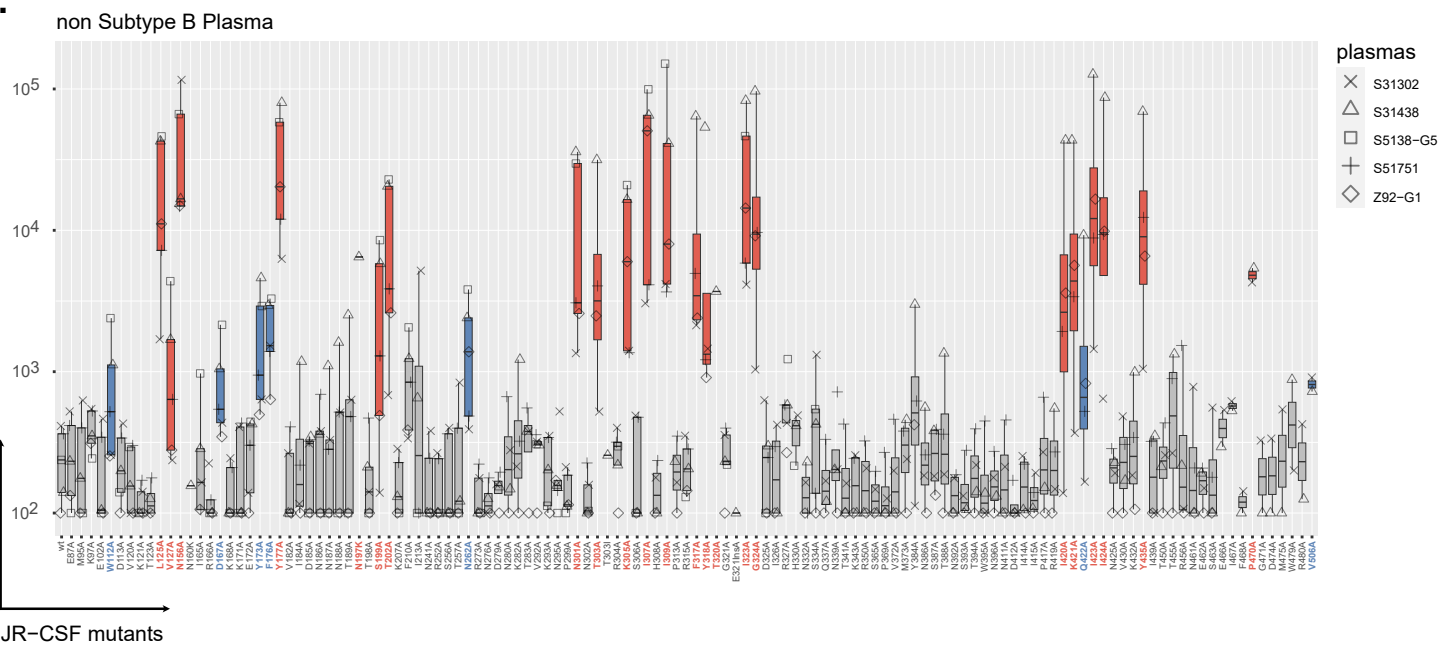

Supplement: S1 Fig — The general neutralization sensitivity of 126 JR-CSF envelope (Env) mutant pseudoviruses was determined in a TZM-bl based assay using A. a set of 16 PWH plasma from chronic infection (eleven subtype B infected, five with non-B subtypes). B. ID50s for a subset of 5/16 plasma that did not inhibit JR-CSF wt Env at an inhibitory dilution ID50 = 100, the minimal dilution probed (four plasma samples from subtype B, one from non-subtype B infected individuals). C. ID50s for five plasma samples from PWH chronically infected with non-subtype B HIV-1 (subtype of infection in parentheses). Three plasma samples neutralized JR-CSF wt, two did not. (Boxplots show center line: median; box limits extend from the 25th to 75th percentiles; whiskers indicate minimum and maximum values). Mutants with high and moderate general neutralization sensitivity as assigned by the analysis presented in Fig 2A are colored in red and blue respectively. S2 Table provides an overview of the plasma samples used. Titrations of each plasma on all viruses was done once, except for the JR-CSF wt reference (n = 4). (PDF) [file ppat.1012825.s001.pdf]

S2 Fig

A.

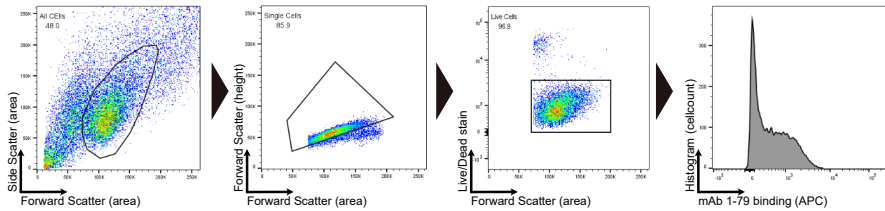

B.

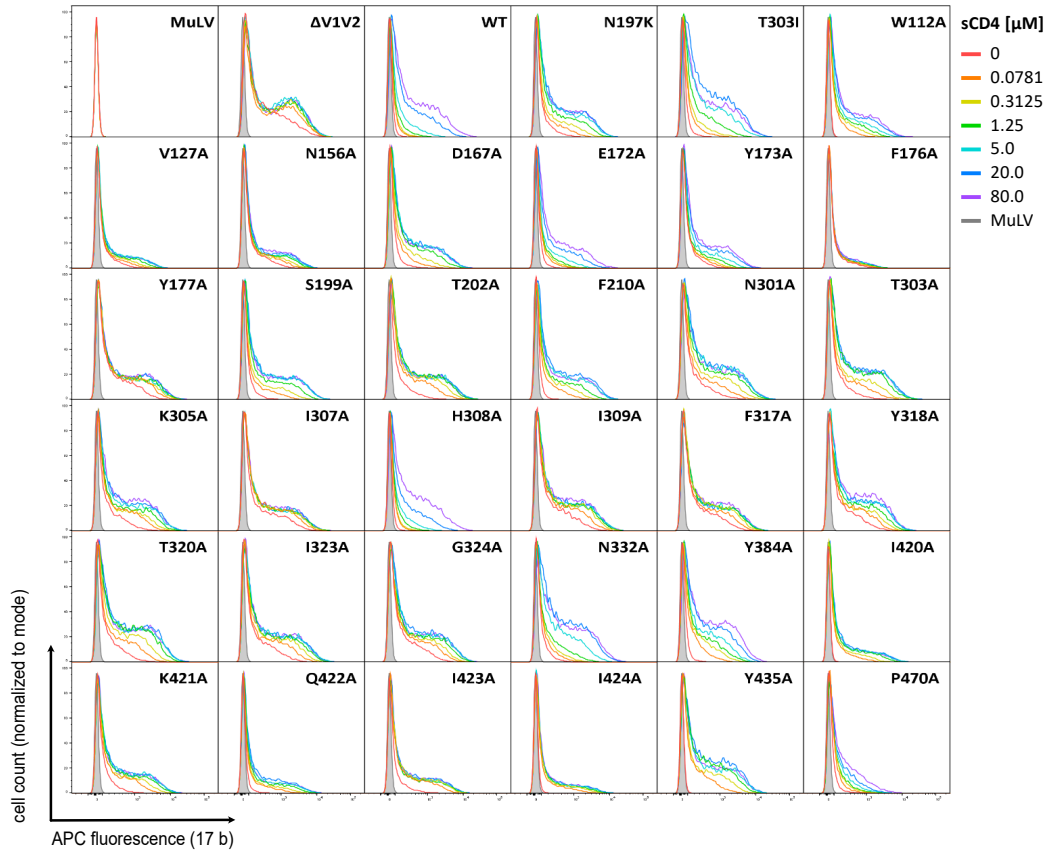

C.

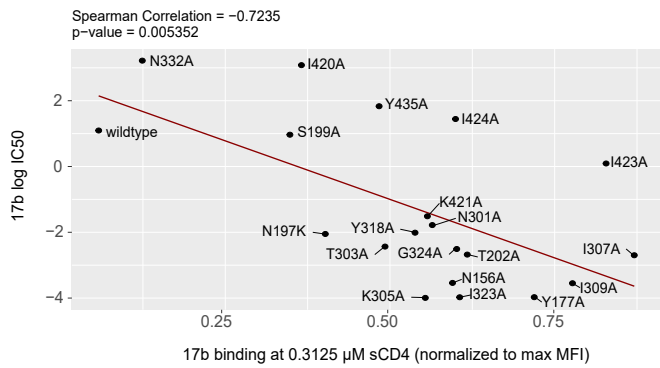

D.

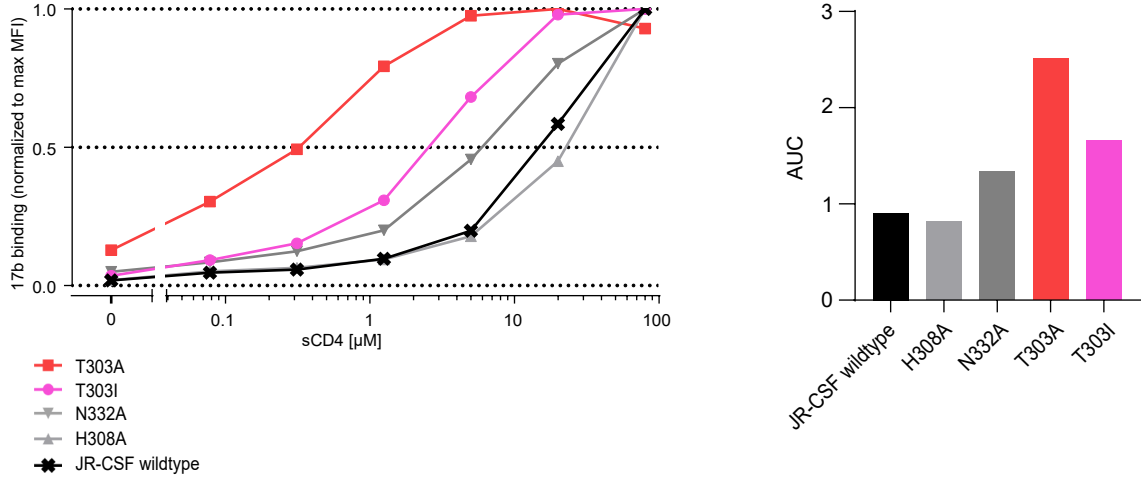

Supplement: S2 Fig — Opening of 293T cell surface expressed Envs by increasing concentrations of sCD4-183 is monitored by flow cytometry after staining with mAb 17b, directed against a CD4-induced epitope similar to the CCR5 co-receptor binding site. A. Example to illustrate the gating strategy applied during analysis by flow cytometry. B. Histograms showing the 17b staining obtained for each Env mutant. C. Spearman correlation between 17b neutralization sensitivity of JR-CSF wildtype and mutant viruses and 17b binding according to Fig 4B. In comparison to Fig 4C mutations directly affecting 17b binding [65] were included. D. Env mutants T303A and T303I are differentiated by 17b binding at baseline, i.e., in absence of sCD4, and by their propensity to expose the 17b epitope with increased concentrations of sCD4-183. The bar graph on the right shows corresponding area under the MFI curve values. All titrations were performed once. (PDF) [file ppat.1012825.s002.pdf]

S3 Fig

A.

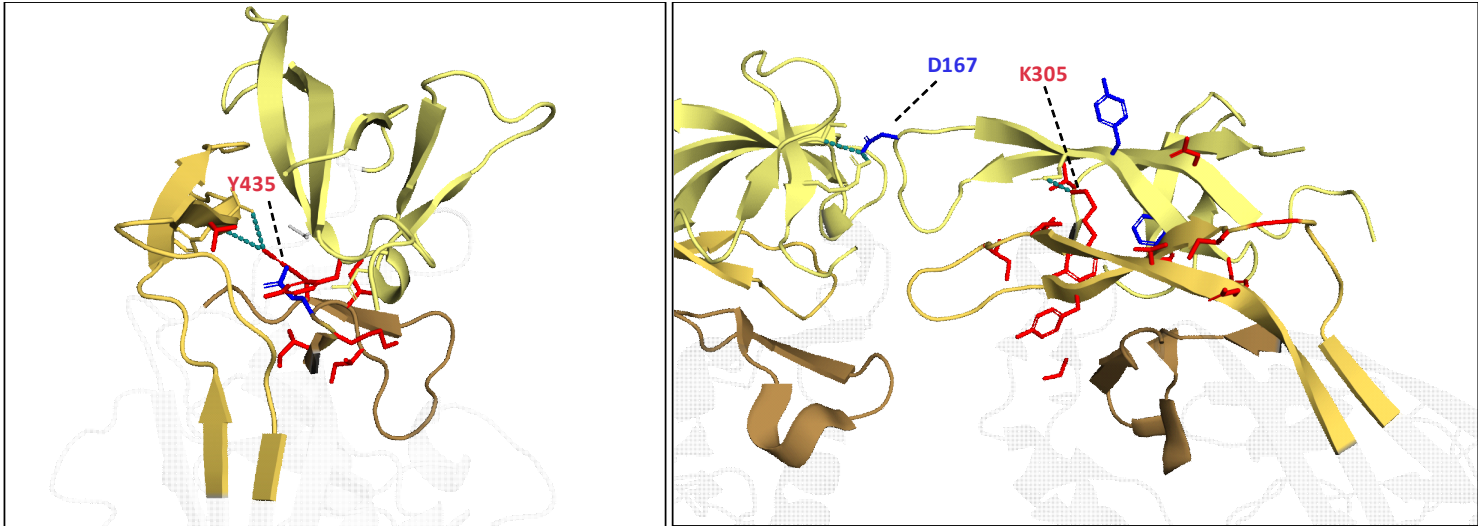

B.

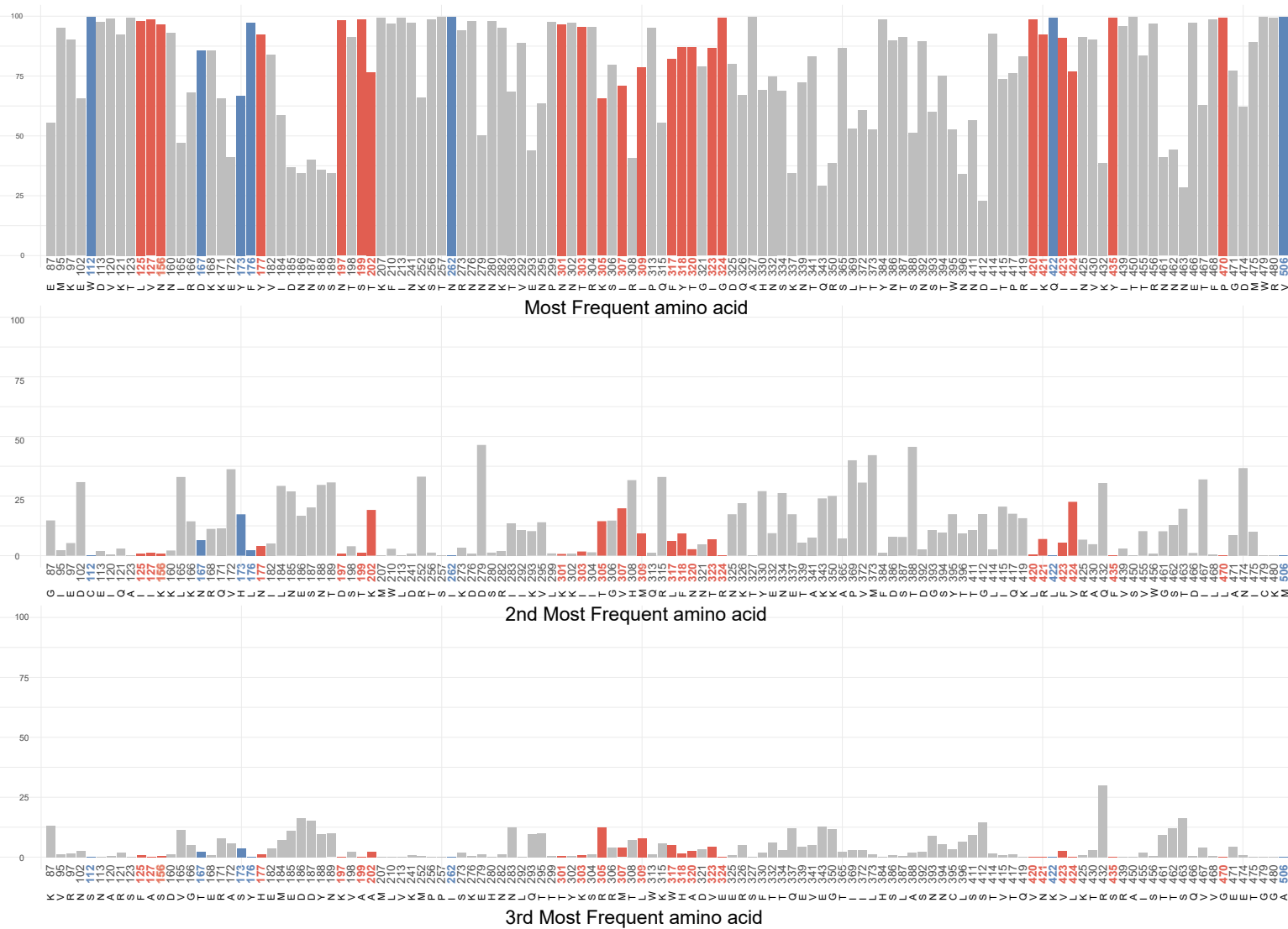

Supplement: S3 Fig — A. Alanine-substitutions leading to moderate (blue) or high (red) generalized neutralization sensitivity of the JR-CSF Env were mapped onto the trimeric closed prefusion structure of the closely related JR-FL Env ectodomain (PDB: 5FYK; V1V2: yellow, V3: orange, β20-β21: brown, gp120: light grey, gp41: dark grey). The enlargements show interactions identified in the structure mediated by the side chains of amino acids critical for neutralization sensitivity (D167 in V2: interprotomer salt bridges to T128 and R192 in V2; K305 in V3: intraprotomer salt bridge to E172 in V2; Y435 in β20-β21: intraprotomer hydrogen-bonds to Y318 and T319 in V3). B. Conservation of all 125 amino-acid positions mutated in the full JR-CSF Env mutant panel. A superfiltered web alignment (available from the Los Alamos National Laboratory database, http://www.lanl.com) of diverse HIV-1 Env sequences (6084 Env sequences from group M including CRFs) was downloaded for the most recent year available (2021) and all relevant amino acid positions were analyzed for their conservation. Residues associated with high (red) and moderate (blue) general neutralization sensitivity are indicated. (PDF) [file ppat.1012825.s003.pdf]

S4 Fig

A.

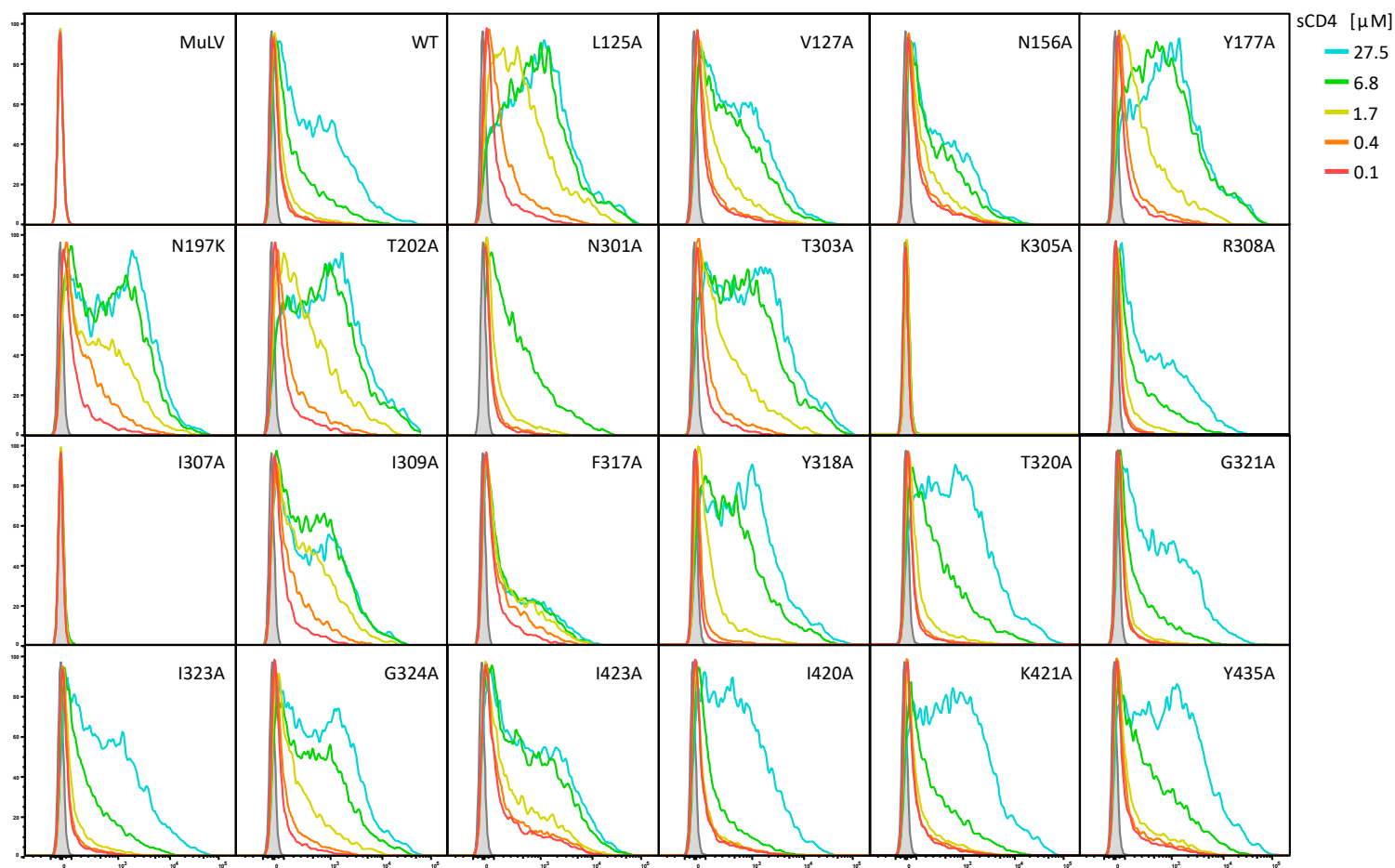

B.

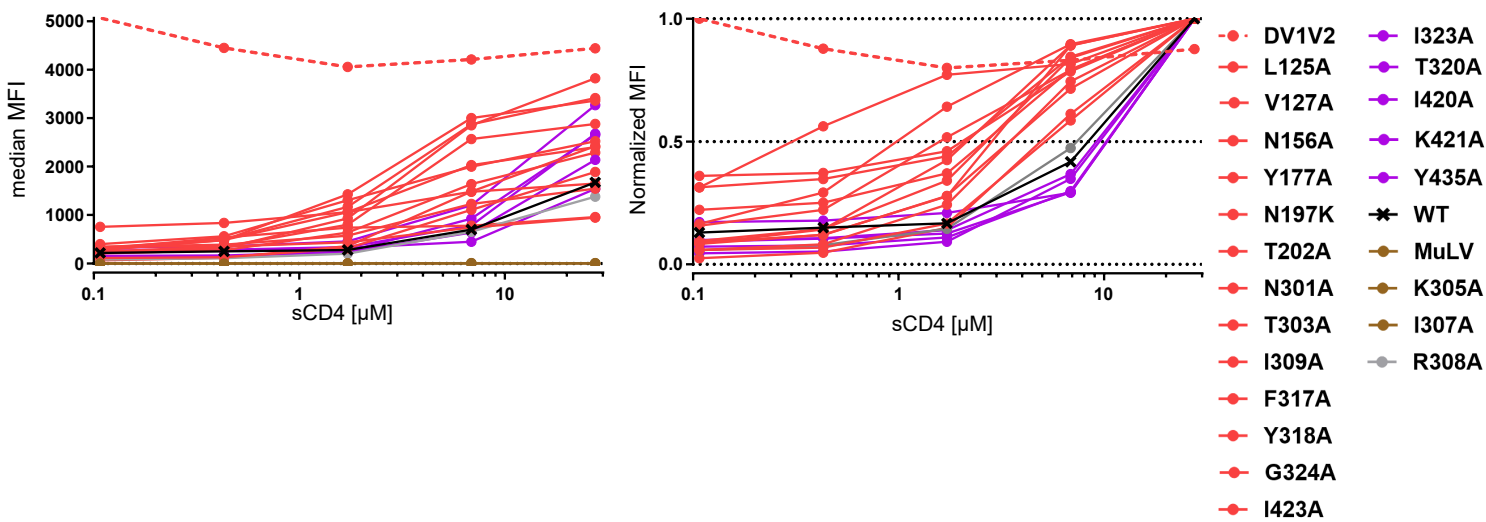

Supplement: S4 Fig — Titration of 293T cell surface expressed BG505 wt envelope and mutants with sCD4-183. The induced opening of the Env trimer was monitored with V3-crown directed mAb 1–79 and analyzed by FACS. A. Histograms showing mAb 1–79 staining. B. Dose-response curves for all Envs tested. The reference envelope BG505_T332N and the R308A mutant (the corresponding JR-CSF mutant has a wt-like phenotype) are colored in black and grey, respectively. BG505 mutants in red reproduce the enhanced propensity to adopt the CD4i state observed for corresponding JR-CSF mutants, the mutants in purple do not. A V1V2 deleted BG505 T332N envelope was included as control (dashed red line). K305A and I307A are 1–79 knock-out mutants (brown). MuLV envelope was used as control for unspecific staining (brown). The two panels show the same data without (left) and with (right) normalization to the highest signal obtained for each mutant respectively. (PDF) [file ppat.1012825.s004.pdf]

S5 Fig

A.

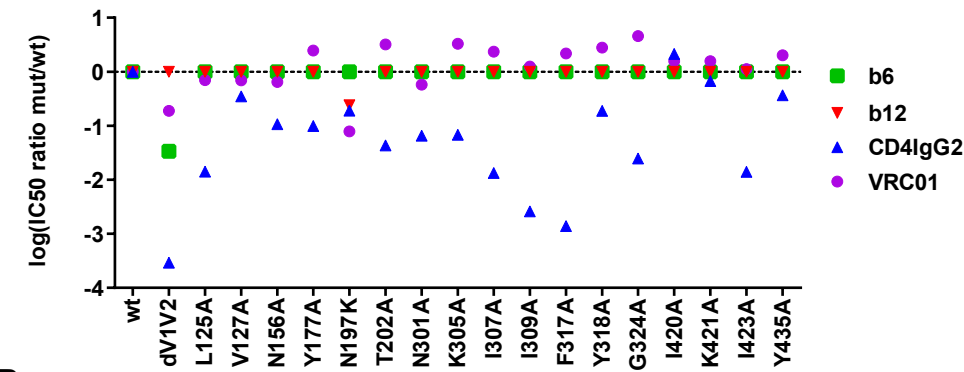

B.

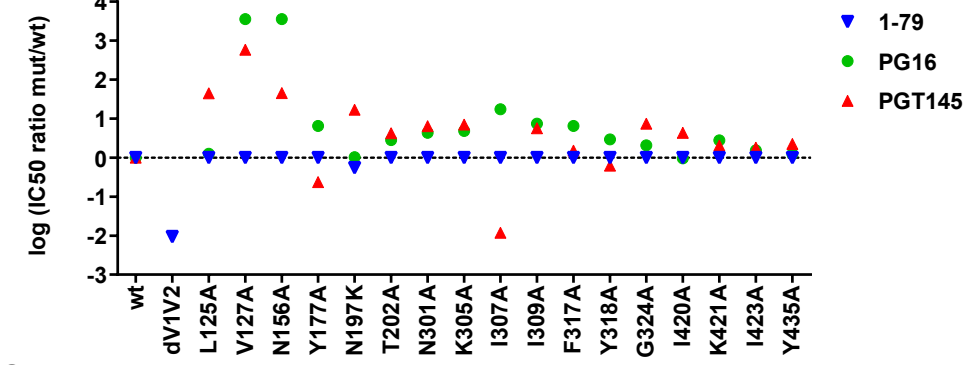

C.

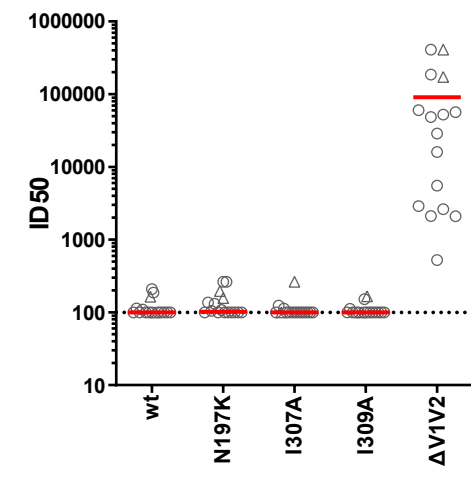

Supplement: S5 Fig — Neutralization sensitivity was determined for 17 alanine mutants based on the BG505 T332N Env (designated as wt) corresponding to JR-CSF alanine mutants with strong general neutralization sensitive phenotype using a pseudovirus assay system with TZM-bl cells. A. and B. Plots show changes in IC50 values relative to the wt envelope. A BG505 T332N Env lacking V1V2 (ΔV1V2) was included as a sensitive control. Depicted are results using A. CD4bs-directed, B. V3-crown directed (1–79), and V2-glycan directed (PG16, PGT145) Abs. C. ID50 values were determined for three BG505 Env mutants using 16 PWH plasma samples (13 infected with subtype B (circles) and 3 with non-subtype B (triangles) HIV). The minimal dilution of plasma tested was 1/100. For each mutant the median ID50 is indicated with a red bar. (PDF) [file ppat.1012825.s005.pdf]

S6 Fig

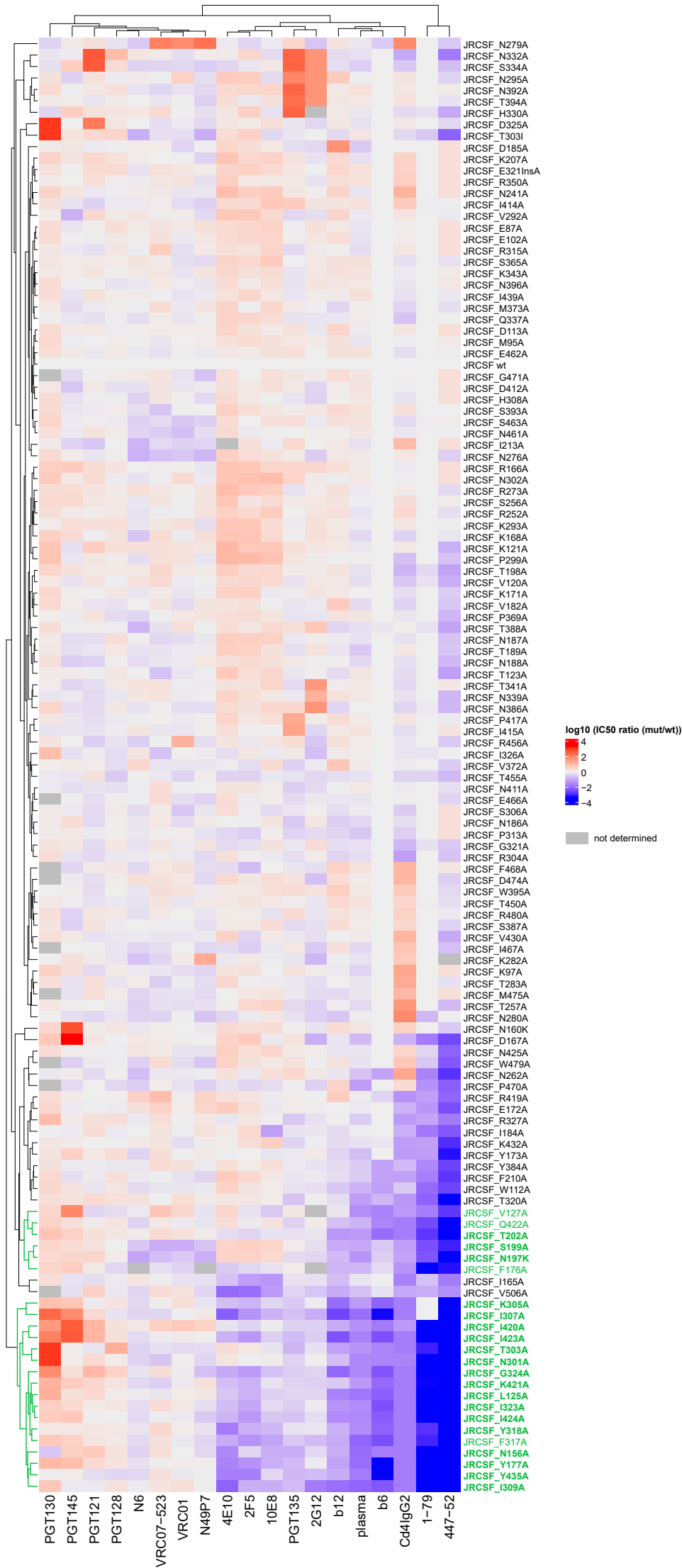

Supplement: S6 Fig — All 126 JR-CSF mutant Envs included in the full virus panel were grouped according to their changes in neutralization sensitivity compared to wt (log IC50 ratios (mut/wt)) against the antibodies indicated at the bottom. For PWH plasma the median reciprocal ID50 ratio (mut/wt) over 11 JR-CSF wt neutralizing plasma (Fig 2A) was used. Two clusters of comprising generally neutralization sensitive mutants are highlighted in green. Mutants in bold were selected for the SENSE-19 virus panel. (PDF) [file ppat.1012825.s006.pdf]

## S7 Fig

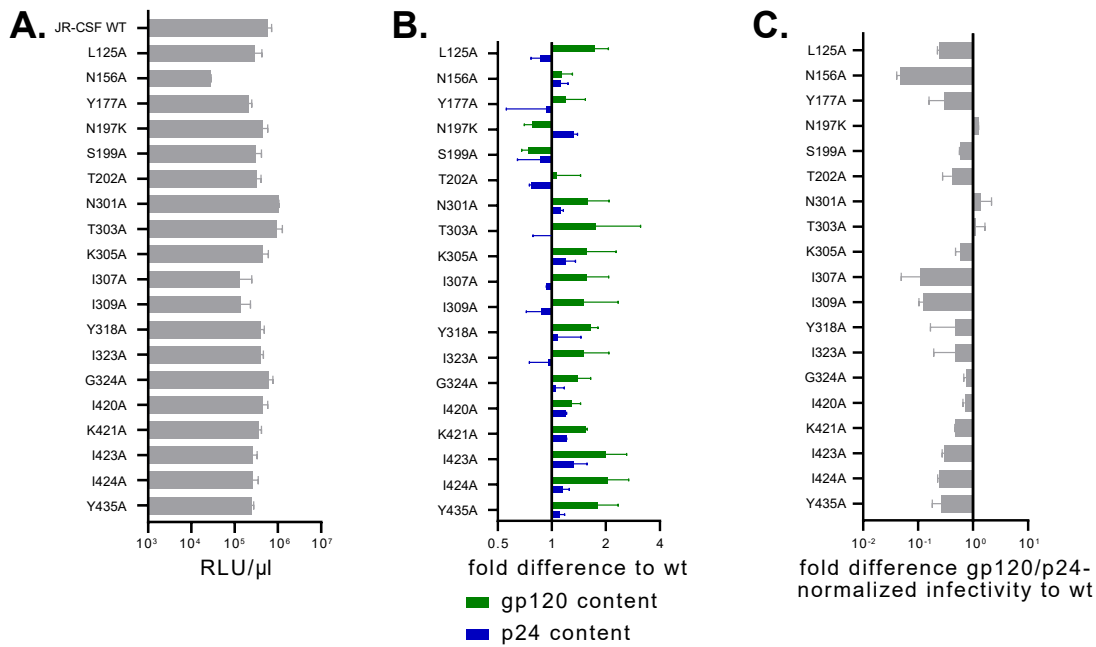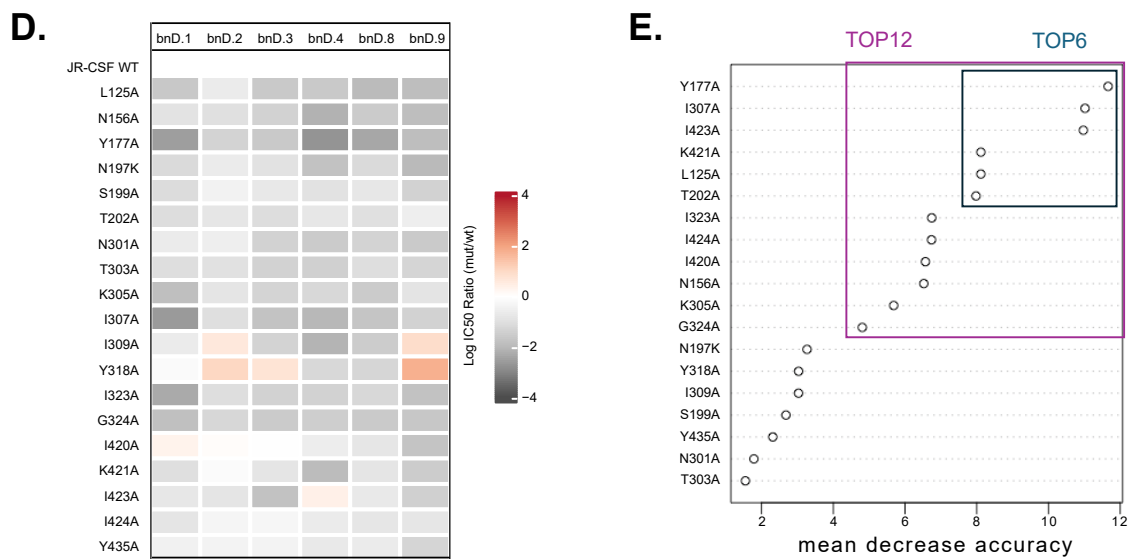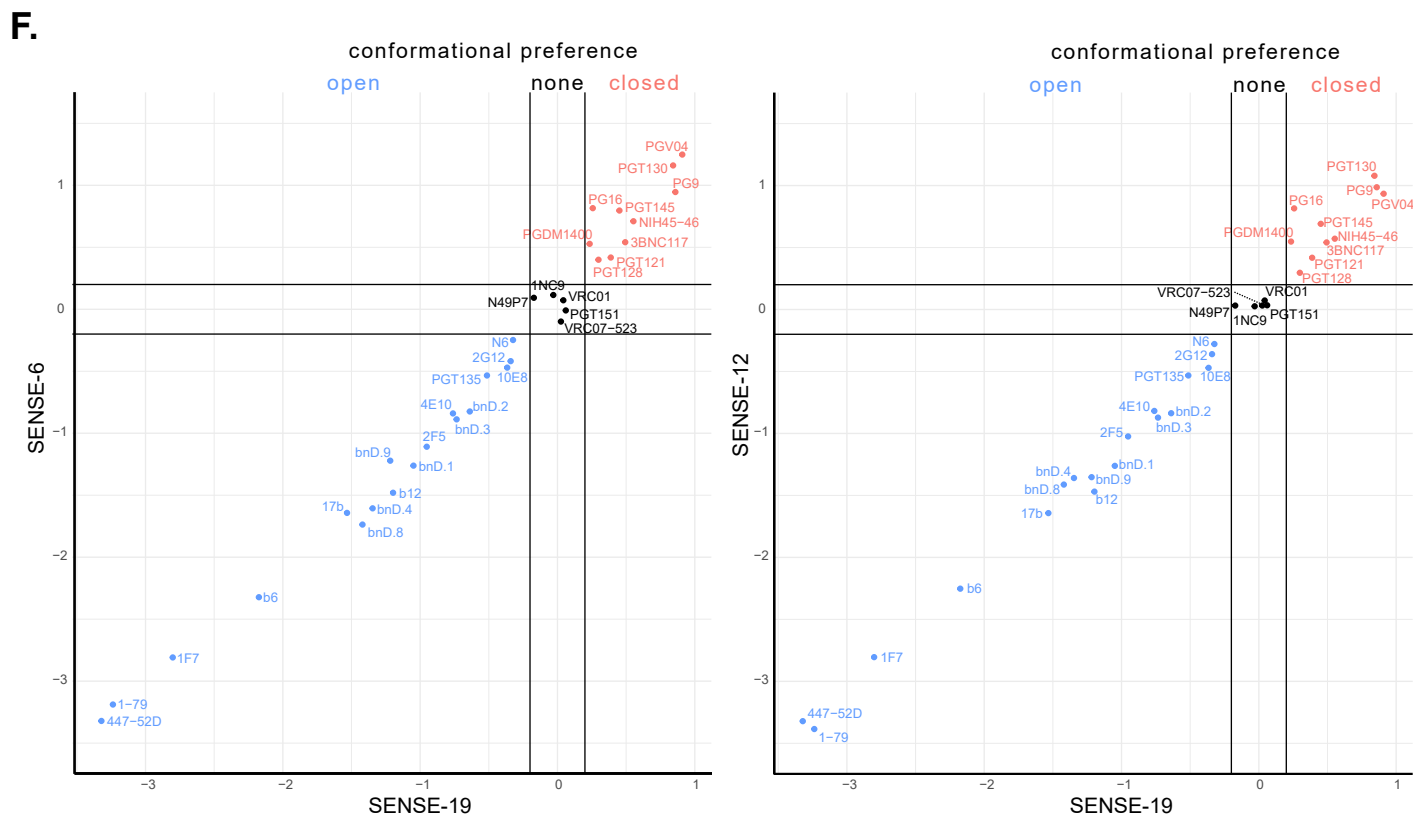

Supplement: S7 Fig — Infectivity analysis of SENSE-19 pseudoviruses. A. Two independent transfections were done for each pseudovirus and each stock was titrated in duplicate on TZM-bl cells. B. The same stocks as in (A) were used to determine the gp120 and p24 content by ELISA. Each sample was titrated in duplicate. Data is expressed relative to pseudovirus with wildtype JR-CSF Env. C. Infectivity shown in (A) was normalized to gp120/p24 content shown in (B) and expressed relative to pseudovirus with wildtype JR-CSF Env. D. Neutralization fingerprinting of monovalent bnDs on the SENSE-19 mutant virus panel. IC50 ratios (mutant/wt) are displayed as a heatmap. E. Random forest analysis on all SENSE-19 mutants using data shown in Fig 5A. The analysis assesses the contribution of each mutant to the classification of bnAbs into the three categories of conformational dependence shown in Fig 5C. The combination of 6 (TOP 6), respectively 12 mutants (TOP 12) that yielded the best classification are highlighted by boxes. F. Comparison of SENSE-19 scores with scores obtained with the TOP 6 and TOP 12 mutant panels determined in (E). (PDF) [file ppat.1012825.s007.pdf]

S8 Fig

A.

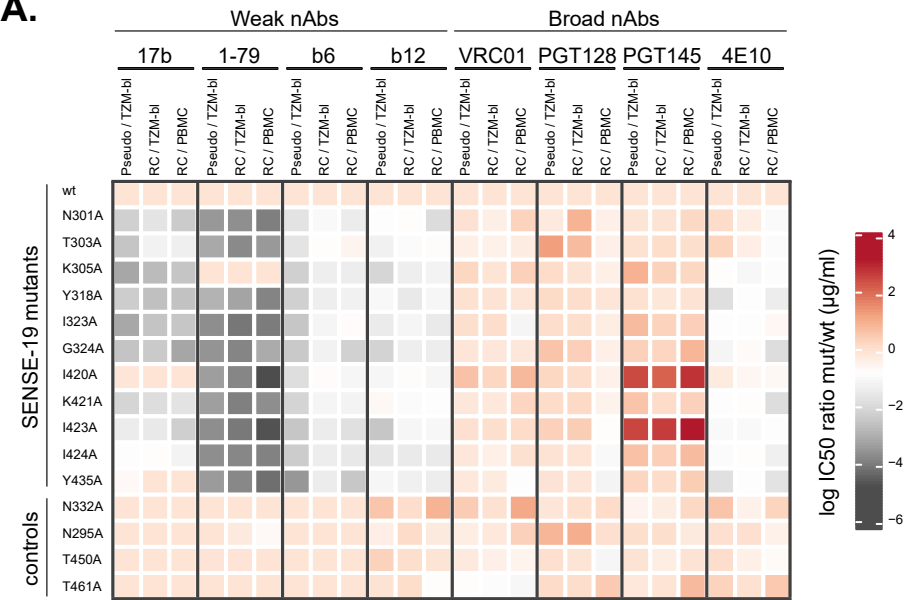

B.

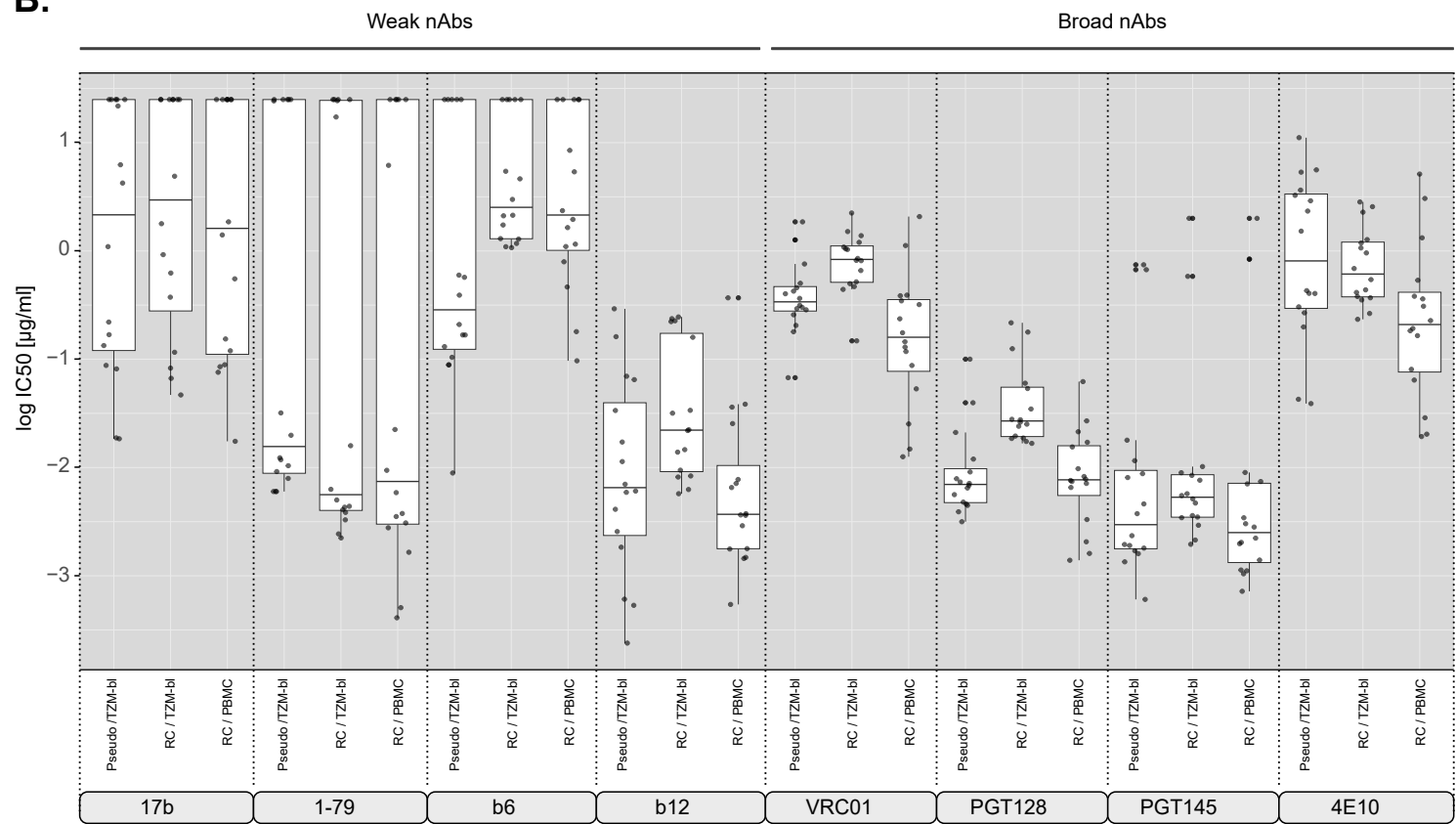

Supplement: S8 Fig — JR-CSF wt virus and Env mutants with and without high overall neutralization sensitivity were tested in three different assay formats. IC50 values were determined in a pseudovirus assay system with TZM-bl reporter cells and in two set-ups with replication competent virus (JR-CSFrc) infecting either TZM-bl cells or PBMCs. A. Heatmap of log IC50 ratio (mut/wt) values. B. Distribution of IC50 values corresponding to data in A. Box plot indicates median (center line), 25th to 75th percentiles (box limits) as well as minima and maxima (whiskers). Titrations in the pseudovirus assay were performed once, except for the JR-CSF wt reference (n ≥ 4). Ab titrations in the JR-CSFrc/TZM-bl assay were set up in triplicates. For replication competent virus on PBMCs results are shown from one of two independent experiments set-up in triplicate for each mAb respectively. (PDF) [file ppat.1012825.s008.pdf]

S9 Fig

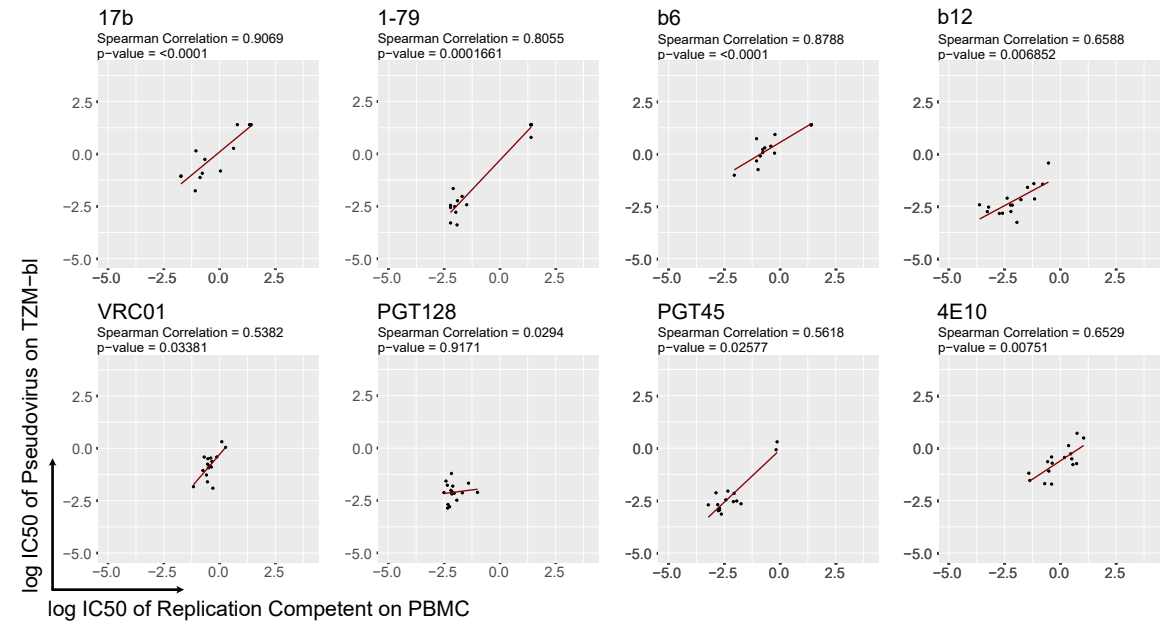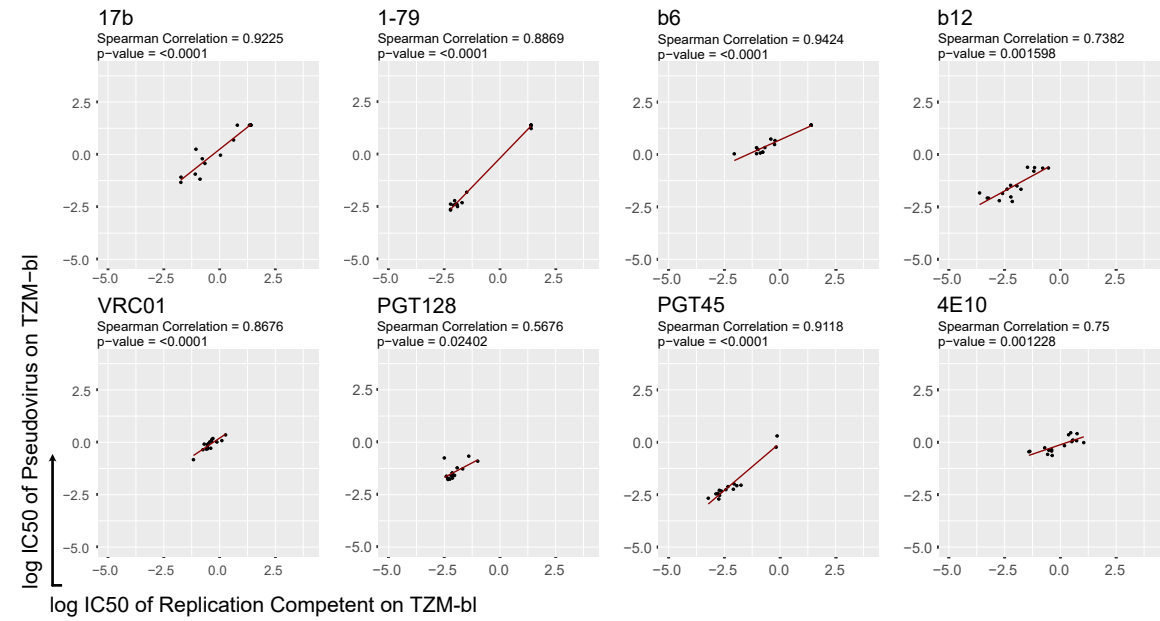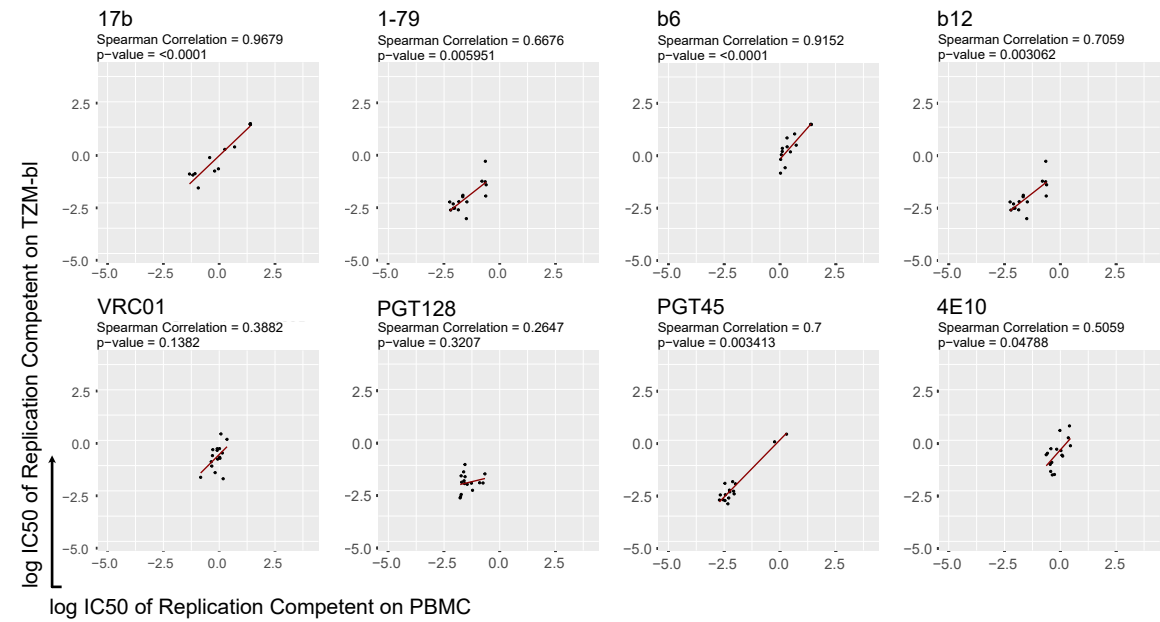

Supplement: S9 Fig — Log IC50 values were correlated for the Abs indicated from two of three different assay formats respectively, based on data shown in Fig 6A. (PDF) [file ppat.1012825.s009.pdf]

S10 Fig

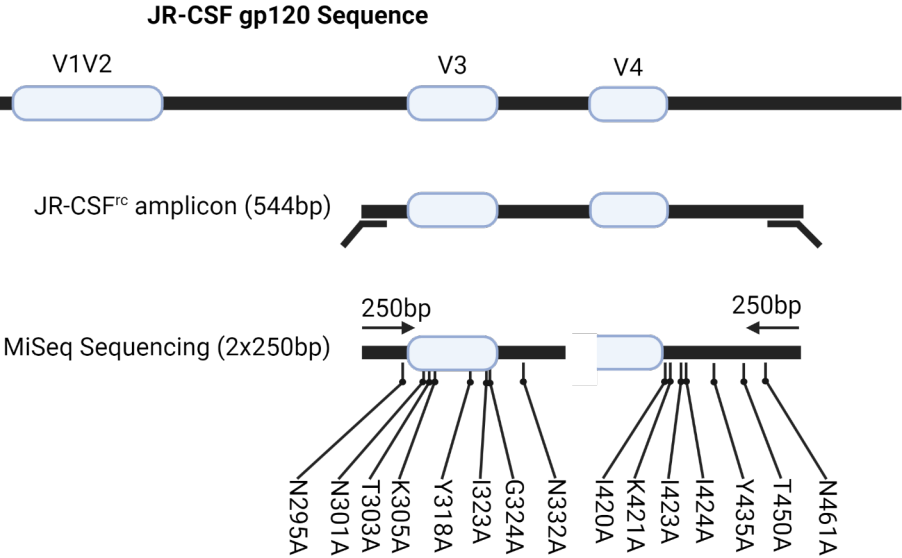

Supplement: S10 Fig — The schematic indicates the positioning of primer binding sites used to determine the composition of a replication competent virus mixture containing JR-CSF wt and point mutated envs by Illumina MiSeq sequencing. (PDF) [file ppat.1012825.s010.pdf]
