# Supplementary material for: Assessing bnAb potency in the context of HIV-1 envelope conformational plasticity
Source: PLoS Pathog. 2025 Jan 21;21(1):e1012825. doi: 10.1371/journal.ppat.1012825 (PMC11774494; doi:10.1371/journal.ppat.1012825)
Supplement: S1 Table — (PDF) [file ppat.1012825.s011.pdf]

**S1 Table**

| | CATNAP neutralization data per Tier-category | | | | | |
|---|---|---|---|---|---|---|
| | 3 | 2 or 3 | 2 | 1B or 2 | 1B | 1A |
| 1-79:IC50 | 0 | 1 | 3 | 1 | 3 | 2 |
| 10E8:IC50 | 84 | 12 | 364 | 17 | 33 | 12 |
| 17b:IC50 | 8 | 17 | 153 | 19 | 12 | 9 |
| 1F7:IC50 | 1 | 1 | 28 | 2 | 1 | 2 |
| 2F5:IC50 | 85 | 22 | 392 | 19 | 37 | 15 |
| 2G12:IC50 | 88 | 22 | 384 | 19 | 35 | 14 |
| 3BNC117:IC50 | 31 | 12 | 291 | 17 | 28 | 12 |
| 447-52D:IC50 | 7 | 17 | 161 | 18 | 14 | 11 |
| 4E10:IC50 | 85 | 22 | 388 | 19 | 37 | 15 |
| BG18:IC50 | 1 | 8 | 108 | 8 | 2 | 2 |
| CD4-Ig:IC50 | 8 | 16 | 135 | 18 | 8 | 8 |
| N49P7:IC50 | 16 | 12 | 185 | 16 | 15 | 9 |
| N6:IC50 | 31 | 11 | 261 | 15 | 27 | 11 |
| NIH45-46:IC50 | 10 | 12 | 165 | 16 | 14 | 7 |
| PG16:IC50 | 59 | 15 | 253 | 16 | 21 | 12 |
| PG9:IC50 | 84 | 15 | 372 | 17 | 36 | 13 |
| PGDM1400:IC50 | 31 | 12 | 302 | 17 | 28 | 11 |
| PGT121:IC50 | 83 | 12 | 369 | 17 | 35 | 14 |
| PGT128:IC50 | 32 | 12 | 309 | 17 | 31 | 13 |
| PGT130:IC50 | 1 | 9 | 115 | 9 | 5 | 5 |
| PGT135:IC50 | 6 | 12 | 167 | 16 | 11 | 8 |
| PGT145:IC50 | 32 | 12 | 302 | 17 | 28 | 11 |
| PGT151:IC50 | 7 | 12 | 184 | 16 | 13 | 10 |
| VRC-PG04:IC50 | 6 | 12 | 159 | 16 | 10 | 7 |
| VRC01:IC50 | 87 | 20 | 392 | 19 | 37 | 13 |
| VRC07-523-LS:IC50 | 31 | 11 | 252 | 15 | 25 | 9 |
| b12:IC50 | 88 | 22 | 394 | 19 | 36 | 14 |
| max number of strains per tier-category | 88 | 22 | 415 | 19 | 38 | 16 |
