# Supplementary material for: Assessing bnAb potency in the context of HIV-1 envelope conformational plasticity
Source: PLoS Pathog. 2025 Jan 21;21(1):e1012825. doi: 10.1371/journal.ppat.1012825 (PMC11774494; doi:10.1371/journal.ppat.1012825)
Supplement: S2 Table — (PDF) [file ppat.1012825.s012.pdf]

S2 Table

Plasma donor	JR-CSF neutralizing dataset	JR-CSF non-neut	non-B data	VL	Subtype	CD4	weeks after infection	weeks off ART
Z07-G3	x			19,000	B	313	304	193
Z40-G1		x		33,400	B	NA	123	123
Z36-G3	x			16,000	B	363	272	180
Z92-G1		x	x	8,600	AE	277	277	221
Z38-G1	x			2,250	B	279	142	142
Z39-G3	x			74,000	B	328	258	174
Z91-G5	x			10,004	B	359	345	345
Z49-G3	x			59,000	B	408	250	197
Z48-G1	x			91,800	B	258	127	127
Z62-G3		x		56,752	B	352	282	227
Z75-G3		x		69,000	B	376	159	159
Z71-G1		x		125,530	B	217	71	71
Z02-G1	x			181,462	B	388	108	108
S31438	x		x	7,130	A	NA	NA	NA
S51751			x	101,000	C	NA	NA	NA
S31302	x		x	54,500	C	NA	NA	NA
S5138-G5			x	85,700	AE	NA	NA	NA
S52611	x			1,551	B	NA	271	62
