# Supplementary material for: Assessing bnAb potency in the context of HIV-1 envelope conformational plasticity
Source: PLoS Pathog. 2025 Jan 21;21(1):e1012825. doi: 10.1371/journal.ppat.1012825 (PMC11774494; doi:10.1371/journal.ppat.1012825)
Supplement: S3 Table — (PDF) [file ppat.1012825.s013.pdf]

S3 Table

Name	Epitope	Reference	Source of expression plasmid	Source of protein
b12	CD4-bs	1	D. Burton, The Scripps Research Institute, La Jolla, USA	H. Katinger and D. Katinger, Polymun, Vienna, Austria
b6	CD4-bs	1	D. Burton, The Scripps Research Institute, La Jolla, USA	H. Katinger and D. Katinger, Polymun, Vienna, Austria
1F7	CD4-bs	2		H. Katinger and D. Katinger, Polymun, Vienna, Austria
3BNC117	CD4-bs	3	M. Nussenzweig, The Rockefeller University, New York, USA	Own production in 293T cells
PGV04	CD4-bs	4	J. Mascola*, Vaccine Research Center, National Institutes of Health, Bethesda, USA	Own production in 293T cells
VRC01	CD4-bs	4	J. Mascola*, Vaccine Research Center, National Institutes of Health, Bethesda, USA	Own production in 293T cells
NIH45-46	CD4-bs	3	M. Nussenzweig, The Rockefeller University, New York, USA	Own production in 293T cells
1NC9	CD4-bs	3	Custom synthesized	Own production in 293T cells
VRC07-523	CD4-bs	5	Custom synthesized	Own production in 293T cells
N6	CD4-bs	6	Custom synthesized	Own production in 293T cells
N49P7	CD4-bs	7	Custom synthesized	Own production in 293T cells
CD4-IgG2	CD4-bs	8		W. Olson*, Progenics Pharmaceuticals Inc
sCD4	CD4-bs	9, 10		W. Olson*, Progenics Pharmaceuticals Inc
sCD4-183	CD4-bs	11	Custom synthesized	Own production in E.coli
17b	CD4i	12	Custom synthesized	J. Robinson*, Tulane University Medical Center, New Orleans, USA and own production in 293T cells
2G12	High Mannose Patch	13		H. Katinger and D. Katinger, Polymun, Vienna, Austria
PGT151	Interface/Fusionpeptide	14	D. Burton, The Scripps Research Institute, La Jolla, USA	Own production in 293T cells
10E8	MPER	15	M. Connors*, National Institute of Allergy and Infectious Diseases, NIH, Bethesda, USA	Own production in 293T cells
4E10	MPER	16		H. Katinger and D. Katinger, Polymun, Vienna, Austria
2F5	MPER	2		H. Katinger and D. Katinger, Polymun, Vienna, Austria
PGT145	V2-Glycan	17	D. Burton, The Scripps Research Institute, La Jolla, USA	Own production in 293T cells
PG9	V2-Glycan	18	D. Burton, The Scripps Research Institute, La Jolla, USA	Own production in 293T cells
PG16	V2-Glycan	18	D. Burton, The Scripps Research Institute, La Jolla, USA	Own production in 293T cells
PGDM1400	V2-Glycan	19	D. Burton, The Scripps Research Institute, La Jolla, USA	Own production in 293T cells
PGT121	V3 High Mannose Patch	17	D. Burton, The Scripps Research Institute, La Jolla, USA	Own production in 293T cells
PGT128	V3 High Mannose Patch	17	D. Burton, The Scripps Research Institute, La Jolla, USA	Own production in 293T cells
PGT130	V3 High Mannose Patch	17	D. Burton, The Scripps Research Institute, La Jolla, USA	Own production in 293T cells
PGT135	V3 High Mannose Patch	17	D. Burton, The Scripps Research Institute, La Jolla, USA	Own production in 293T cells
BG18	V3 High Mannose Patch	20	Custom synthesized	Own production in 293T cells
447-52D	V3-crown	21	S. Zolla-Pazner (Icahn School of Medicine at Mount Sinai, New York, NY, USA)	H. Katinger and D. Katinger, Polymun, Vienna, Austria
1-79	V3-crown	22	M. Nussenzweig, The Rockefeller University, New York, USA	Own production in 293T cells
bnD.1	V3-crown	23	Friedrich et al. 2021 Nat Commun. 2021; 12(1):6705.	Own production in E.coli
bnD.2	V3-crown	23	Friedrich et al. 2021 Nat Commun. 2021; 12(1):6705.	Own production in E.coli
bnD.3	V3-crown	23	Friedrich et al. 2021 Nat Commun. 2021; 12(1):6705.	Own production in E.coli
bnD.4	V3-crown	24	Glögl et al. 2023 Nat Struct Mol Biol. 2023; 30(9):1323-1336.	Own production in E.coli
bnD.8	αV3C	24	Glögl et al. 2023 Nat Struct Mol Biol. 2023; 30(9):1323-1336.	Own production in E.coli
bnD.9	αV3C	24	Glögl et al. 2023 Nat Struct Mol Biol. 2023; 30(9):1323-1336.	Own production in E.coli
* Through the NIH AIDS Reagent Program, Division of AIDS, NIAID, NIH				

References

1 Barbas CF, 3rd, Bjorling E, Chiodi F, Dunlop N, Cababa D, Jones TM, et al. Recombinant human Fab fragments neutralize human type 1 immunodeficiency virus in vitro. Proc Natl Acad Sci U S A. 1992;89(19):9339-43. Epub 1992/10/01. PubMed PMID: 1384050; PubMed Central PMCID: PMC50122.

2 Buchacher A, Predl R, Strutzenberger K, Steinfellner W, Trkola A, Purtscher M, et al. Generation of human monoclonal antibodies against HIV-1 proteins; electrofusion and Epstein-Barr virus transformation for peripheral blood lymphocyte immortalization. AIDS Res Hum Retroviruses. 1994;10(4):359-69. doi: 10.1089/aid.1994.10.359. PubMed PMID: 7520721.

3 Scheid JF, Mouquet H, Ueberheide B, Diskin R, Klein F, Oliveira TY, et al. Sequence and structural convergence of broad and potent HIV antibodies that mimic CD4 binding. Science. 2011;333(6049):1633-7. Epub 2011/07/19. doi: 10.1126/science.1207227. PubMed PMID: 21764753.

4 Wu X, Yang ZY, Li Y, Hogerkorp CM, Schief WR, Seaman MS, et al. Rational design of envelope identifies broadly neutralizing human monoclonal antibodies to HIV-1. Science. 2010;329(5993):856-61. Epub 2010/07/10. doi: 10.1126/science.1187659. PubMed PMID: 20616233; PubMed Central PMCID: PMC2965066.

5 Rudicell RS, Kwon YD, Ko SY, Pegu A, Louder MK, Georgiev IS, et al. Enhanced potency of a broadly neutralizing HIV-1 antibody in vitro improves protection against lentiviral infection in vivo. J Virol. 2014;88(21):12669-82. Epub 20140820. doi: 10.1128/JVI.02213-14. PubMed PMID: 25142607; PubMed Central PMCID: PMCPMC4248941.

6 Huang J, Kang BH, Ishida E, Zhou T, Griesman T, Sheng Z, et al. Identification of a CD4-Binding-Site Antibody to HIV that Evolved Near-Pan Neutralization Breadth. Immunity. 2016;45(5):1108-21. doi: 10.1016/j.immuni.2016.10.027. PubMed PMID: 27851912; PubMed Central PMCID: PMCPMC5770152.

7 Sajadi MM, Dashti A, Rikhtegaran Tehrani Z, Tolbert WD, Seaman MS, Ouyang X, et al. Identification of Near-Pan-neutralizing Antibodies against HIV-1 by Deconvolution of Plasma Humoral Responses. Cell. 2018;173(7):1783-95 e14. Epub 20180503. doi: 10.1016/j.cell.2018.03.061. PubMed PMID: 29731169; PubMed Central PMCID: PMCPMC6003858.

8 Allaway GP, Davis-Bruno KL, Beaudry GA, Garcia EB, Wong EL, Ryder AM, et al. Expression and characterization of CD4-IgG2, a novel heterotetramer that neutralizes primary HIV type 1 isolates. AIDS Res Hum Retroviruses. 1995;11(5):533-9. Epub 1995/05/01. PubMed PMID: 7576908.

9 Deen KC, McDougal JS, Inacker R, Folena-Wasserman G, Arthos J, Rosenberg J, et al. A soluble form of CD4 (T4) protein inhibits AIDS virus infection. Nature. 1988;331(6151):82-4. Epub 1988/01/07. doi: 10.1038/331082a0. PubMed PMID: 3257544.

10 Fisher RA, Bertonis JM, Meier W, Johnson VA, Costopoulos DS, Liu T, et al. HIV infection is blocked in vitro by recombinant soluble CD4. Nature. 1988;331(6151):76-8. Epub 1988/01/07. doi: 10.1038/331076a0. PubMed PMID: 2829022.

11 Ivan B, Sun Z, Subbaraman H, Friedrich N, Trkola A. CD4 occupancy triggers sequential pre-fusion conformational states of the HIV-1 envelope trimer with relevance for broadly neutralizing antibody activity. PLoS Biol. 2019;17(1):e3000114. Epub 20190116. doi: 10.1371/journal.pbio.3000114. PubMed PMID: 30650070; PubMed Central PMCID: PMCPMC6351000.

12 Thali M, Moore JP, Furman C, Charles M, Ho DD, Robinson J, et al. Characterization of conserved human immunodeficiency virus type 1 gp120 neutralization epitopes exposed upon gp120-CD4 binding. J Virol. 1993;67(7):3978-88. Epub 1993/07/01. PubMed PMID: 7685405; PubMed Central PMCID: PMC237765.

13 Trkola A, Dragic T, Arthos J, Binley JM, Olson WC, Allaway GP, et al. CD4-dependent, antibody-sensitive interactions between HIV-1 and its co-receptor CCR-5. Nature. 1996;384(6605):184-7. Epub 1996/11/14. doi: 10.1038/384184a0. PubMed PMID: 8906796.

14 Falkowska E, Le KM, Ramos A, Doores KJ, Lee JH, Blattner C, et al. Broadly neutralizing HIV antibodies define a glycan-dependent epitope on the prefusion conformation of gp41 on cleaved envelope trimers. Immunity. 2014;40(5):657-68. Epub 20140424. doi: 10.1016/j.immuni.2014.04.009. PubMed PMID: 24768347; PubMed Central PMCID: PMCPMC4070425.

15 Huang J, Ofek G, Laub L, Louder MK, Doria-Rose NA, Longo NS, et al. Broad and potent neutralization of HIV-1 by a gp41-specific human antibody. Nature. 2012;491(7424):406-12. Epub 2012/11/16. doi: 10.1038/nature11544. PubMed PMID: 23151583.

16 Stiegler G, Kunert R, Purtscher M, Wolbank S, Voglauer R, Steindl F, et al. A potent cross-clade neutralizing human monoclonal antibody against a novel epitope on gp41 of human immunodeficiency virus type 1. AIDS Res Hum Retroviruses. 2001;17(18):1757-65. Epub 2002/01/15. doi: 10.1089/08892220152741450. PubMed PMID: 11788027.

17 Walker LM, Huber M, Doores KJ, Falkowska E, Pejchal R, Julien JP, et al. Broad neutralization coverage of HIV by multiple highly potent antibodies. Nature. 2011;477(7365):466-70. Epub 2011/08/19. doi: 10.1038/nature10373. PubMed PMID: 21849977; PubMed Central PMCID: PMC3393110.

18 Walker LM, Phogat SK, Chan-Hui PY, Wagner D, Phung P, Goss JL, et al. Broad and potent neutralizing antibodies from an African donor reveal a new HIV-1 vaccine target. Science. 2009;326(5950):285-9. Epub 2009/09/05. doi: 10.1126/science.1178746. PubMed PMID: 19729618.

19 Sok D, van Gils MJ, Pauthner M, Julien JP, Saye-Francisco KL, Hsueh J, et al. Recombinant HIV envelope trimer selects for quaternary-dependent antibodies targeting the trimer apex. Proc Natl Acad Sci U S A. 2014;111(49):17624-9. Epub 20141124. doi: 10.1073/pnas.1415789111. PubMed PMID: 25422458; PubMed Central PMCID: PMCPMC4267403.

20 Freund NT, Wang H, Scharf L, Nogueira L, Horwitz JA, Bar-On Y, et al. Coexistence of potent HIV-1 broadly neutralizing antibodies and antibody-sensitive viruses in a viremic controller. Sci Transl Med. 2017;9(373). doi: 10.1126/scitranslmed.aal2144. PubMed PMID: 28100831; PubMed Central PMCID: PMCPMC5467220.

21 Gorny MK, Conley AJ, Karwowska S, Buchbinder A, Xu JY, Emini EA, et al. Neutralization of diverse human immunodeficiency virus type 1 variants by an anti-V3 human monoclonal antibody. J Virol. 1992;66(12):7538-42. Epub 1992/12/01. PubMed PMID: 1433529; PubMed Central PMCID: PMC240465.

22 Scheid JF, Mouquet H, Feldhahn N, Seaman MS, Velinzon K, Pietzsch J, et al. Broad diversity of neutralizing antibodies isolated from memory B cells in HIV-infected individuals. Nature. 2009;458(7238):636-40. Epub 2009/03/17. doi: 10.1038/nature07930. PubMed PMID: 19287373.

23 Friedrich N, Stiegeler E, Glögl M, Lemmin T, Hansen S, Kadelka C, et al. Distinct conformations of the HIV-1 V3 loop crown are targetable for broad neutralization. Nat Commun. 2021;12(1):6705. Epub 20211118. doi: 10.1038/s41467-021-27075-0. PubMed PMID: 34795280; PubMed Central PMCID: PMCPMC8602657.

24 Glögl M, Friedrich N, Cerutti G, Lemmin T, Kwon YD, Gorman J, et al. Trapping the HIV-1 V3 loop in a helical conformation enables broad neutralization. Nat Struct Mol Biol. 2023;30(9):1323-36. Epub 20230821. doi: 10.1038/s41594-023-01062-z. PubMed PMID: 37605043; PubMed Central PMCID: PMCPMC10497408.
