# Supplementary material for: Assessing bnAb potency in the context of HIV-1 envelope conformational plasticity
Source: PLoS Pathog. 2025 Jan 21;21(1):e1012825. doi: 10.1371/journal.ppat.1012825 (PMC11774494; doi:10.1371/journal.ppat.1012825)
Supplement: S4 Table — (PDF) [file ppat.1012825.s014.pdf]

S4 Table

clade	virus strain	Genbank entry	Tier
A	BG505_W6M_C2_T332N	DQ208458	2
	KER2018.11	AY736810	2
	MG505.W0M.ENV.A2	DQ208449	2
	Q23_17	AF004885	1B
	Q769_H5	AF407159	2
	Q842_D12	AF407160	2
B	ZEnv16_1202_7	KU600818	2
	ZEnv07_0504_15	KU600814	2
	ZEnv91_0505_12	KU600815	2
	BaL_26	DQ318211	1B
	JR-CSF	AY669726	2
	JR-FL	AY669728	2
	NAB5pre_cl_1	EU023923	2
	NAB9pre_cl_106	EU023928	2
	PVO.04	AY835444	2
	QH0692, clone 42	AY835439	2
	REJO4541 clone 67	AY835449	2
	RHPA4259 clone 7	AY835447	2
	TRO clone 11	AY835445	2
	WITO4160 clone 33	AY835451	2
C	25925_2_22	EF117273	1B
	CAP45.2.00.G3	DQ435682	2
	CAP88_6mo.c10	KU198436	2
	Du156.12	DQ411852	2
	DU422.1	AY043175	2
	ZM106F.PB9	AY424163	2
	ZM214M.PL15	DQ3885	2
	ZM233M.PB6	DQ388517	2
	ZM249M.PL1	DQ388514	2
	ZM53_12	AY423984	2
G	T252_7	EU513190	2
	NAB13pre_cl_9	EU023937	2
	X2088_c9	EU885764	2
CRF01_AE	ZEnv32_0111_5	KU600816	2
	ZEnv92_1008_8	KU600817	2
	C1080.c3	JN944660	2
	CNE5	HM215415	2
	CNE59	HM215422	2
CRF02_AG	T250_4	EU513189	2
CRF07_BC	CNE40	HM215414	1B
