# Supplementary material for: Assessing bnAb potency in the context of HIV-1 envelope conformational plasticity
Source: PLoS Pathog. 2025 Jan 21;21(1):e1012825. doi: 10.1371/journal.ppat.1012825 (PMC11774494; doi:10.1371/journal.ppat.1012825)
Supplement: S5 Table — (PDF) [file ppat.1012825.s015.pdf]

S5 Table

**PCR 1: Addition of Adapters**			**PCR 2: Barcoding**		
**Reaction mix:**		Vol (µl)	**Reaction mix:**		Vol (µl)
H_2O		33	molecular grade H_2O		12.2
5x Kapa HiFi Buffer		10	5x Kapa HiFi Buffer		4
dNTPs Kapa (10mM)		1.5	dNTPs Kapa (10mM)		0.6
Primer Fwd-JR-CSF (10µM)		1.5	Index Primer 1 (10 µM)		1
Primer Rev-JR-CSF (10µM)		1.5	Index Primer 2 (10 µM)		1
$MgCl_2$ Kapa (25mM)		0.5			
Kapa HiFi enzyme		1	Kapa HiFi enzyme		1
DNA template		1	DNA template		1
**PCR settings:**			**PCR settings:**		
Initial denaturation	95°C, 3 min		Initial denaturation	95°C, 5 min	
Denaturation	98°C, 20 sec	30 cycles	Denaturation	98°C, 20 sec	17 cycles
Primer annealing	65°C, 20 sec		Primer annealing	60°C, 15 sec	
Elongation	72°C, 30 sec		Elongation	72°C, 30 sec	
Final elongation	72°C, 5 min		Final elongation	72°C, 5 min	
Hold	4°C		Hold	4°C	
